# La Grippe—Origin, History, Treatment

**Published:** 1891-11

**Authors:** V. W. Gayle

**Affiliations:** Kansas City, Mo.


ARTICLE II.

### LA GRIPPE—ORIGIN, HISTORY, TREATMENT.

This disease is sometimes called Russian Influenza, because it is supposed to have had its origin in Siberia. It usually occurs as an epidemic and travels rapidly over the globe. It has received many names, the most common being Influenza and La Grippe. The Italians, in the seventeenth century, called it Influenza, supposing it owed its origin to some occult influence of the stars. La Grippe is said by some to come from the Polish *Crypka,* meaning "hoarse;" others recognize it in the French word "gripper," which means "to seize;" the Germans call it "blitz katarrh." It has been known in Europe since the fifth century, and invariably sweeps over the country from east to west. In the recent universal epidemic it travelled from St. Petersburg to New York in about six weeks. Its etiology is shrouded in mystery, although the generally accepted theory is that it is bacterial in its origin. The diplococcus pneumoniæ and the streptococcus pyogenes are the usual varieties of bacteria of the atmosphere. The streptococcus seems to be the species that is usually found to predominate, and in some cases is found alone. I take it that the disease is beyond question infectious, and spreads by atmospheric influences. Many examples are to be found in the current medical literature which could be cited to prove the assumption; also the experience of most physicians during the recent epidemics among their clientele was in this direction. Some investigators claim, however, that it is contagious, and regard it as a contagio-miasmatic disease capable of being conveyed from place to place and from person to person. I am of the opinion, however, that there must be receptivity, else why should some escape?

It attacks persons of all ages and conditions, although children are more apt to be exempt. The micro-organism seems to act specifically upon the mucous membrane of the respiratory tract with which it comes in contact.

Humidity of the atmosphere seems to have much to do with its development. Altitude seems to make little difference. It is seen in the valleys of large rivers and high up in the mountain ranges; under the snows of Scandinavia; under the suns of the Equator, as well as on the islands of Polynesia; in the house and in the palace; in the busy mart and in my lady's boudoir.

Greenley, in the *American Practitioner and News*, of Louisville, Ky., says: "Influenza is independent of hydrometric, thermometric and barometric changes."

Prudden finds certain kind of bacteria. Marmorek, in addition to these, finds several other kinds, and doubts the investigations of Ribbert, while Babes and Kowalski see others which no one else has described. Klebs, in examining the blood, finds abnormal bodies; Kollman finds additional ones, while other bacteriologists report negative results. In my opinion influenza is due to a definite micro-organism which is in the atmosphere under certain conditions and at certain places, which conditions of the atmosphere last for a longer or shorter period. When these micro-organisms come in contact with the mucous-membrane of the respiratory tract, if the conditions are such that they find lodgment, then the disease is manifested. Now, whether the pneumococcus, the streptococcus or the staphlococcus, collectively or individually, is the cause of influenza, I am not able to say, and proof has not been forthcoming from investigators which is reliable and settled. The Pathogenic bacillus of influenza has as yet not been definately recognized, notwithstanding the worthy labors of Tomasi-Crudelli, Klebs and others. My intention is not to condemn the investigations that have taken place in this direction, far be it from me. I am a firm believer in the germ theory of the disease. Some go so far as to say that the disease cannot be caused primarily by a microbe, because of its rapid propagation. This is illogical; all that is necessary is for the individual to be in such a condition that the germ can find receptivity, and the development will then go on. Influenza has been rightly divided into three varieties: First, the neurotic form; second, the catarrhal form; third, the gastric form. There may be combinations of the above varieties.

The temperature noticed in the various localities where this pandemic fever prevailed ranged from 104° to 105° F., and usually remained at this height for two or three days and subsided rapidly, the febrile movement being symptomatic. This disease resembles materially Dengue; both have, in common, sudden invasion, high temperature, lumbar, and muscular pain, with cephalalgia.

It is also analogous to the epizootic that frequently prevails among the equine family. Measles sometimes resembles influenza; particularly when the latter is attended by an eruption. In the surgical wards of hospitals, when influenza is prevailing, the ulcers and wounds often assume a gangrenous character, the result of the infection by the microbe of influenza. There is usually a large number of persons in nearly every community sick of various serious ailments; when influenza is added to their troubles the mortality is much greater than in ordinary cases of either disease alone.

There is one pecularity about this disease that seems to have been little noticed, and that is the baneful influence influenza exerts in some localities on pregnant women. Quite a number of abortions, followed by serious hemorrhage, occurred in several places.

Now, whether this lack of notice is due to neglect of observation or is dependent on climate influences, I do not know. The neurotic type of this trouble consists chiefly of nervous symptoms, fever, prostration, headaches, lumbago, pleurodynia, pains in the limbs, and sometimes a general cutaneous hyperesthesia. Delirium is often noted. Some cases show giddiness and tinnitus aurium, which may last for weeks after the disease has subsided. The olfactory nerves are frequently effected and show perversion of the sense of smell. The auditory apparatus is occasionally involved, in fact, various manifestations of the neuroses show themselves, particularly when there is a neuropathic predisposition.

Pathologically, there seems to be nothing abnormal relating to the influenza *per se*, except it be a lesion of the spinal cord, as has been seen in some post-mortem examinations. The arachnoid membrane has been found severely congested; this obtained all along the spine, in the cervical, dorsal and lumbar regions. Degenerative changes were present in various portions of the cord. Meningitis and also myelitis sometimes occur. Neuralgia of the various nerves are common. There may be muscular atrophy, the result of degeneration in the motor nerve cells.

In the catarrhal or pulmonary form, there is in the outset a sense of chilliness along the spine, in some cases a pronounced chill, with high fever, sore throat, frontal headache, sneezing, pains in the limbs and verious parts of the body, particularly in the thoracic region. Pneumonic symptoms occur in many patients, with frequent cases of pneumonia. Perhaps 40 per cent. of these catarrhal cases of influenza have a typical pneumonia; severe couch, with dyspnea and tightness of the chest, is often present; soreness about the eyes; coryza and all the symptoms of a "bad cold" is the usual order of things. The pulse ranges from 90 to 120, usually full in volume, although in many cases it gets small and wiry. The urine in nearly all types of influenza shows an increase in the acid constitutents; there is often indications of bile, and, in a few cases hematuria. The torpid liver is frequently a concominant, followed of course, by constipation. When pneumonia supervenes in this disease, it rarely reaches the stage of hepatization. There is seldom the pathognomonic prune-juice expectoration. Pulmonary abscess and pleurisy are occasional results. The angina is sometimes very severe, with croupous deposits on the tonsils and pharynx. Laryngitis, with swollen and reddened vocal cords, is often seen. The bronchitis is frequently grave. Ear diseases are in many cases a prominent sequel, particularly otitis media. Influenza being a peculiar essential fever, the bronchitis is the local manifestation of the constitutional trouble, and not primary or idiopathic. There have been some cases in which hemoptysis occurred, where only bronchial signs could be detected, which recovered without further pulmonary trouble, thus showing a total absence of tubercle at the time of the hemorrhage.

In the gastric form of the disease, there is usually nausea and vomiting, with pain seemingly throughout the mucous membrane of the alimentary tract, also the respiratory tract. The vomiting sometimes is persistent; the liver and spleen are involved, and show abnormal conditions. Frequently the pyrexia and concomitant symptoms give you the impression that there is tendency to the typhoid condition, although the characteristic temperature curve of typhoid fever is absent in influenza. Many of these symptoms may be merged into one—e. g., nervous postration; inflammation of the mucous membrance of the respiratory tract and disturbances of the digestive organs may affect the patient at the same time.

Alopecia in some cases follows an attack of influenza. Erythematous and erysipelatous eruptions may occur, and at times are troublesome. Anorexia prevails in nearly every ease. Cystltis and nephritis are sometimes seen and can be traced to the influenza, or rather to the morbific agent producing it; but in senile cases, particularly if complications exist, there is great danger. The mental derangements, insanity, &c., usually pass away after the patient is convalescent. In many cases the convalescence is slow and tedious, and great care must be observed in the management of these patients. Stimulants and restoratives are here absolutely needed. Epidemic influenza, if not properly managed, is more liable to serious complications than nearly any other disease we know of. The nervous symptoms in this disease are remarkable from the fact that they are scarcely ever alike in any two cases. Blocq says: "First. Certain nervous affections are really grippal, in the sense that they may be considered special localization of influenza upon the nervous system. Second. Another group depends equally upon the grippe, but, secondarily, it is not the grippe itself, but the secondary infections which have determined the neuropathies. Third. A third category includes the neuropathies, in the development of which influenza has only been an exciting factor, determining a recurrence of a previous but recovered-from condition, or bringing about a new condition in an individual predisposed."

TREATMENT.

This disease by proper treatment at the beginning of an attack can be so modified as to be almost aborted. If not properly managed, influenza is particularly liable to grave complications, even in mild cases the tendency is towards prostration, and often the nervous shock is such as to materially debilitate the patient. Where there is much angina with acute bronchial irritation, the following is indicated:

| | | | |
|---|---|---|---|
| R | Ammon. chloride | - - | dr. ii |
| | Potassii chloras | - - - | dr. i |
| | Tinct. ferri chloridi | - - | f. dr. ii |
| | Syr. simplex | - - | f. oz ii |
| | Aquæ | - q.s. ft - - | f. oz iv |

M. Sig.—Teaspoonful in sweetened water every four hours, also apply to the throat with mop every three hours.

Quinine is the best germ destroyer we have for the microbe of influenza. During the recent epidemic I aborted quite a number of cases with antikamnia in combination with salol and quinine. The relief obtained by the administration of antikamnia where the cephalalgia was severe as in the majority of my cases, was wonderful. When the pain seemed almost intolerable, I have seen a ten grain dose of antikamnia banish it. The combination spoken of was as follows:

| | | | |
|---|---|---|---|
| R. | Antikamnia | - - - | dr. i |
| | Salol | - - - | dr. ss |
| | Quinia sulph | - - - | dr. i |

M. ft. Capsules No. XXX.

Sig.—One every two hours.

Mustard pediluvia are of great advantage, and a plaster of mustard and lard, one part of the former to two of the latter, applied directly to the chest, answered admirably as a mild counter irritant.

Expectorants are often needed, and antikamnia should be administered with them, thus :

R. Antikamnia - - - dr. i
Syr. senega - - - f. oz. i
Vini ipecac - - - f, dr. iii
Syr. tolutan - - q. s. ft. f. oz. iv
M. Sig.—Teaspoonful every two hours.

The mild chloride of mercury in minimum doses often repeated will be beneficial. The following prescription is a favorit: of mine.

R. Hydrarg. chlo. mite - - gr. i
Sodii bicarb - - - scr. i
Lactopeptine - - - dr. ss
M. ft. Charts No. x.

Sig.—One every hour until all are taken. This to be followed by a full dose of hunyadi janos or rubinat condal water.

The pyrexia can be controlled by the antikamnia and cold sponging. Digitalis or stropanthus are needed in some cases to tone up the heart; large doses of quinine are advocated by some, but I have never seen the necessity of giving large doses. What is mostly needed is an antithermic analgesic to relieve the pain and reduce the fever. These properties are found in antikamnia. This with the germ destroyer quinia, is all that I really needed in the treatment of this disease. I advocate the use of stimulants in nearly every case. They are frequently needed in the onset of the disease. Sprays of carbolic acid, turpentine or resorcine are frequently efficacious in the laryngeal troubles. The diet should be light and easily digestible. As a diaphoretic, the use of the muriate of pilocarpine has been advocated. I have no experience with it. By careful attention and avoidance of exposure, together with the line of treatment mapped out, the vast majority of cases will recover. Of course, there are occasional cases which present symptoms which require other remedial agents, but these of necessity must be left to the discretion of the medical attendant.—V. W. GAYLE, M. D., Kansas City, Mo., in *Medical World*.

